# Green and Healthier Alternatives to Chemical Additives as Cheese Preservative: Natural Antimicrobials in Active Nanopackaging/Coatings

**DOI:** 10.3390/polym13162675

**Published:** 2021-08-10

**Authors:** Rayssa Cruz Lima, Anna Paula Azevedo de Carvalho, Carla P. Vieira, Rodrigo Vilela Moreira, Carlos Adam Conte-Junior

**Affiliations:** 1 Department of Biochemistry, Chemistry Institute, Federal University of Rio de Janeiro, Rio de Janeiro 21941598, RJ, Brazil; rayssa-cruz01@hotmail.com (R.C.L.); carlavieira@edu.unirio.br (C.P.V.); 2 Center for Food Analysis (NAL), Technological Development Support Laboratory (LADETEC), Federal University of Rio de Janeiro, Rio de Janeiro 21941598, RJ, Brazil; 3 Graduate Program in Chemistry (PGQu), Chemistry Institute, Federal University of Rio de Janeiro, Rio de Janeiro 21941909, RJ, Brazil; 4 Graduate Program in Food Science (PPGCAL), Chemistry Institute, Federal University of Rio de Janeiro, Rio de Janeiro 21941909, RJ, Brazil; 5 Graduate Program in Veterinary Hygiene (PPGHV), Faculty of Veterinary Medicine, Fluminense Federal University (UFF), Niterói 24230340, RJ, Brazil; rodrigovilela@id.uff.br; 6 Graduate Program in Sanitary Surveillance (PPGVS), National Institute of Health Quality Control, Oswaldo Cruz Foundation (FIOCRUZ), Rio de Janeiro 21040900, RJ, Brazil

**Keywords:** cheese shelf-life, biopackaging materials, biobased materials, polyphenols, edible coatings, biopolymers, edible films, nano-antimicrobials, polysaccharides, antimicrobial coatings

## Abstract

The side effects and potential impacts on human health by traditional chemical additives as food preservatives (i.e., potassium and sodium salts) are the reasons why novel policies are encouraged by worldwide public health institutes. More natural alternatives with high antimicrobial efficacy to extend shelf life without impairing the cheese physicochemical and sensory quality are encouraged. This study is a comprehensive review of emerging preservative cheese methods, including natural antimicrobials (e.g., vegetable, animal, and protist kingdom origins) as a preservative to reduce microbial cheese contamination and to extend shelf life by several efforts such as manufacturing ingredients, the active ingredient for coating/packaging, and the combination of packaging materials or processing technologies. Essential oils (EO) or plant extracts rich in phenolic and terpenes, combined with packaging conditions and non-thermal methods, generally showed a robust microbial inhibition and prolonged shelf life. However, it impaired the cheese sensory quality. Alternatives including EO, polysaccharides, polypeptides, and enzymes as active ingredients/nano-antimicrobials for an edible film of coating/nano-bio packaging showed a potent and broad-spectrum antimicrobial action during shelf life, preserving cheese quality parameters such as pH, texture, color, and flavor. Future opportunities were identified in order to investigate the toxicological effects of the discussed natural antimicrobials’ potential as cheese preservatives.

## 1. Introduction

Cheese is a dairy matrix of a wide variety and geographic distribution, being produced in different locations worldwide, and the cheese market was projected to reach the US$ 112 billion mark by 2025 [1]. Although cheese is a fundamental source of proteins considered to have high biological value and high digestibility, its nutritional and physical-chemical characteristics favor the growth and development of microorganisms, reducing its shelf life [2,3,4]. The addition of sodium chloride, potassium sorbate, sodium acetate, sodium benzoate, and other chemical additives during the preparation of matrix products is carried out in order to increase the sensory characteristics (such as flavor and texture) in addition to slowing microbial development and, consequently, extending the shelf life [5]. However, these chemical additives can be harmful to the consumer’s health when ingested at a level above the acceptable daily intake and the recommended daily intake for chemical salt-rich chemical preservatives [6], as daily sodium intake above 2 g is related to the progressive increase in blood pressure and cardiovascular disease (CVD) risks [6,7]. Thus, global measures aimed at daily sodium intake reduction have been encouraged. Accordingly, the World Health Organization (WHO) total daily sodium consumption should be reduced to 1.5 g until 2025—only 30% of the 5 g of salt per day recommended [8]. Moreover, consumers have a more significant concern for healthier manufactured foods, including lower, replaced, and reformulated sodium options [9]. Besides, several researchers reported that too much sodium consumptionmight lead to hypertension and cardiovascular disease risk [6]. Indeed, the food industry recognized its responsibility relative to the sodium content in foods, and several initiatives have been started [8,10]. 

A second reason motivating the study of natural antimicrobials is that they may be an alternative to microbial resistance caused by synthetic antibiotics [11]. However, reducing salt content in cheese can interfere with its microbiological and sensory quality [5]. Thus, the dairy industry has sought technological alternatives that present the same antimicrobial action as traditional preservatives without negatively affecting the product’s sensory quality.

**Figure 1:**
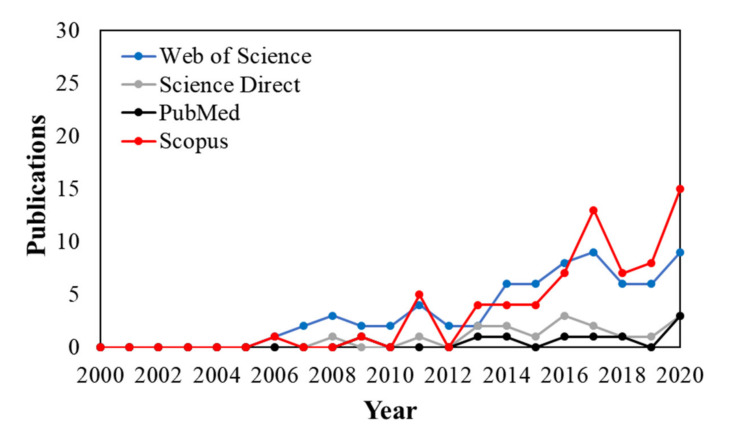
The publications per year according to the research database’s string search: ((“natural antimicrobials” OR “essential oils” OR “bioactive compounds”) AND (packaging OR coating OR nanopackaging) AND cheese).

Figure 1 shows that several scientific publications related to natural antimicrobial-based approaches and nanopackagings/coatings applications in the context of cheese preservation rapidly raised starting from the last decade. Several efforts with essential oils and plant extracts have been performed to preserve cheese, and some of them focus on enhancing raw milk preservation [12] and its role during cheesemaking as an alternative food additive [13,14] due to the presence of several bioactive compounds such as phenolics and terpenes with broad-spectrum antimicrobial activity. On the other hand, in the last decade, edible film coatings in food packaging [15,16] also have become a reality in commercial applications of cheese preservation in order to ensure quality and safety in the industry since they can reduce weight loss, prevent cheese spoilage, and control respiratory rate [17,18,19,20]. In addition, edible films/coatings were also very recently examined as the most widely used system for delivering essential oils/extracts as potential preservatives and antioxidants in cheese [21]. Therefore, this promising strategy has been prospected to possess continuous growth in the future, enhanced by novel and emerging alternatives from nanotechnology [22] such as polymers as the matrix for nanodispersions/nanocomposites to form edible coatings for food preservation [23]. In this view, polysaccharides as alginates [19] and chitosan-based nanocomposite films and coatings were recently highlighted as biopolymers with antimicrobial activities and film-forming properties that are attractive for the packaging/preservation food/dairy industry [24].

However, there are still uncertainties in the natural antimicrobials’ applicability in terms of cheese preservative purposes that might be playing a role in the dairy industry’s current challenges: whether natural antimicrobials can inhibit microbial growth in a manner equal to or more effective than traditional chemical additives in preserving the cheese; and whether it can be applied as antimicrobial agents in order to extend the shelf life comparing its performance with traditional additives in manufacturing. 

Therefore, our study proposed a comprehensive review of the main spoilage/pathogenic microorganisms in cheese reported worldwide, the natural antimicrobials of several origins beyond plants and their primary mechanisms of action of phytochemicals associated, and its usage to reduce microbial count and to extend the shelf life of cheese without impairing cheese quality by several attempts (e.g., as a manufacturing additive, the active ingredient for coating/packaging, and combined methods with packaging conditions or processing technologies). 

## 2. Literature Review and Data Collection

### Microbial Growth Inhibition Analysis

Whenever possible, we report here in this review the dosage values in terms of minimum inhibitory concentration (MIC), minimum fungicide concentration (MFC), or minimum bactericidal concentration (MBC) values reported in the reviewed articles. The percentage values of microbial growth inhibition were obtained in this review by calculating the percentage of microbial count reduction (CR) (expressed by colony-forming units, CFU) in the cheese containing natural antimicrobial compared to the cheese control by an in situ assay, according to Equation (1).

(1) $$\%\;CR=\frac{\left(CFU_{natural\;antimicrobial}-CFU_{control}\right)}{CFU_{control}}\times \;100$$

We also considered studies that showed at least the in vitro antimicrobial activity when an in situ assay with a cheese model was not performed. Thus, when the control sample result was available, the growth inhibition results were shown as a percentage of inhibitory zone relative to the control (RIZ) by data extracted from the reviewed articles, according to Equation (2). When the test with the control sample was not realized, we directly extracted the inhibitory zone (IZ) values reported in the articles from natural antimicrobials samples.

(2) $$\%\;RIZ=\frac{\left(IZ_{natural\;antimicrobial}-IZ_{control}\right)}{IZ_{control}}\times \;100$$

## 3. How Unsuitable Is the Use of Traditional Chemical Additives Now Compared to Cheese Preservation?

Table 1 illustrates the antimicrobial effect of traditional chemical preservatives used to extend the shelf life in cheese. It can be observed that the use of low doses of potassium sorbate (0.1%) [25] and sodium benzoate (0.001%) [26] resulted in no effect/worse effect in the shelf life of cheese [25], although 0.15% ultimately reduced the in vitro *Listeria monocytogenes* counts relative to the control after 60 days of storage at low pH [27].

Recently, the combination treatments as modified atmosphere packaging (MAP) and pulsed light positively affected the shelf life of active packaging/coating of cheese samples. The combined effect of active coating and MAP on prolonging the shelf life and ensure safety under abusive thermal conditions of low-moisture Mozzarella cheese was demonstrated [28]. While uncoated samples were unacceptable from the sensory aspect at 78 days of the microbiological test, the total viable counts only increased up to unacceptable values at the end of storage (160 days) in the PS/sodium alginate-active coated samples [28]. Low doses of sodium benzoate in cheese cheddar slices (packaged by starch films with pulsed light) showed an inhibition effect 28% higher than the control against *Listeria innocua*, despite it being lower than found by citric acid (natural antimicrobial isolated from citric fruits) when combined with pulsed light [26]. These results indicated that higher concentrations of these salts might be needed for a better antimicrobial effect.

### Potential Harmful Effects of Sodium Benzoate and Potassium Sorbate on Human Health

Although benzoate and sorbate salts are generally recognized as safe (GRAS) by the The United States Food and Drug Administration (USFDA), the side effects and potential impacts on human health by their use as food preservatives have been reported as a reason for concern by several researchers. Sodium benzoate was related to skin allergies [29], risk of chronic disease increased by oxidative stress in human erythrocyte in vitro [30], and activation of inflammatory pathways contributing to cancer development [31]. The carcinogenic risk of SB in beverages is also a concern due to its ability to convert to benzene [32]. Furthermore, the consumption of high contents of SB in beverages was also linked with attention deficit hyperactivity disorder (ADHD) symptoms in children [33] and college students [34]. Potassium sorbate showed genotoxicity, mutagenicity, and DNA-damaging activity at high concentrations or combined with nitrites in vivo [35,36] and in vitro against human peripheral blood lymphocytes [37]. Thus, alternative options such as natural antimicrobial agents with significant antimicrobial efficacy to extend shelf life in cheese are needed and have received researchers’ significant attention. 

## 4. Green Preservatives by Natural Antimicrobials as Cheese Preservative

### 4.1. Mechanisms of Action of Antimicrobials from Natural Origins

Before discussing the antimicrobial effect of leading groups of natural antimicrobials able to inhibit the spoilage and pathogenic microbial development and growth in cheese, it is essential to understand, in a general manner, what the primary cellular mechanisms of action are. Among the different microbial metabolic signaling pathways reported to natural compounds, the most common toward natural antimicrobials include membrane permeability alteration [38,39], alterations of a cell wall structure [40], and alterations in metabolism (proteins and nucleic acids synthesis) by folic acid synthesis inhibition [41]. Then, cedar extract (*Moringa oleifera*) [42], basil EO (*Ocimum basilicum* L.) [43,44], oregano (*Origanum vulgare* L.), and rosemary (*Rosmarinus officinalis*) EOs [45] are just a few examples of natural antimicrobials of plant origin rich in bioactive compounds with bacteriostatic or bactericidal effects in cheese by mechanism routes cited above. 

#### 4.1.1. Plant Extracts

**Figure 2:**
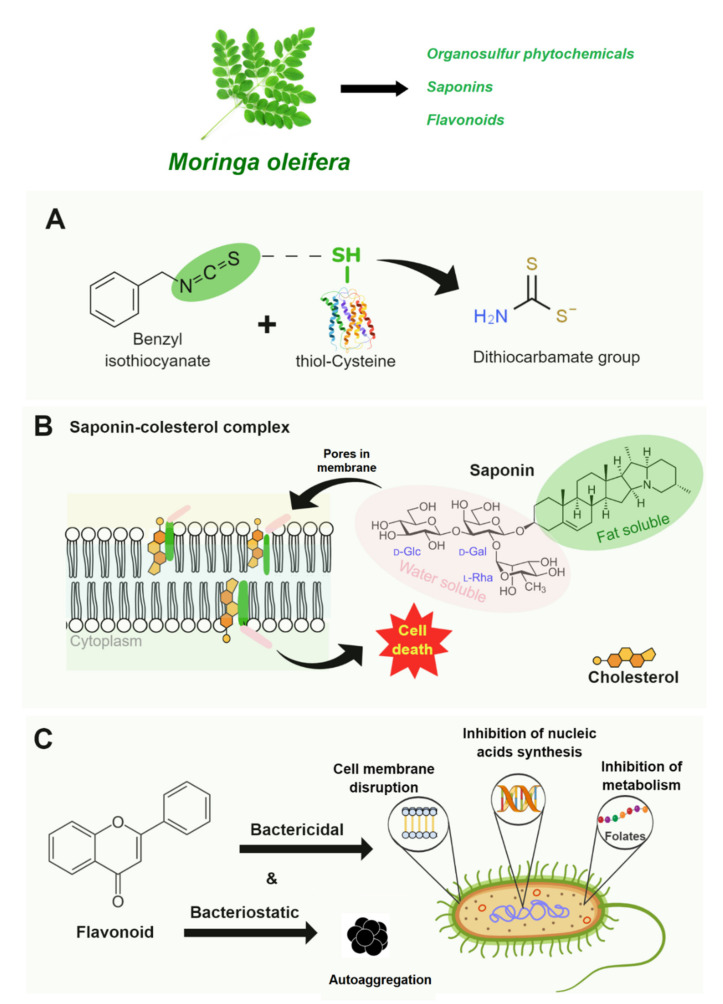
Antimicrobial mechanisms of *Moringa oleifera* bioactive compounds: (**A**) Protein damage-triggered by benzyl isothiocyanates bioconjugation with cysteine. (**B**) Membrane dysfunction-triggered by saponins complex formation with cholesterol. (**C**) Bacteriostatic and bactericidal effects of flavonoids.

Figure 2 shows the antimicrobial mechanisms of bioactive compounds from *M. oleifera*. The *M. oleifera* leaves are rich in varied phytochemicals such as benzyl isothiocyanate (organosulfur phytochemicals), saponins, and flavonoids antimicrobial activity against several pathogens [46,47,48]. The antibacterial activity of BITC, for example, has been linked to protein damage triggered by benzyl isothiocyanates bioconjugation with cysteine, resulting in protein structure changes inactivating microbial growth [49] (Figure 2A). The antifungal activity of saponins has been reported to be linked with membrane dysfunction triggered by saponin’s complex formation with cholesterol, followed by cell death [39] (Figure 2B). On the other hand, the flavonoids can result in bacteriostatic and bactericidal effects [41]: it can promote bacterial cell aggregation (bacteriostatic effect), damage the cell membrane structure, inhibit the enzymatic activities of essential nutrients synthesis, and inhibit energy metabolism by inhibiting the enzymatic activity of the mitochondrial electron transport chain (e.g., NADH-cytochrome c reductase) (bactericidal effect) (Figure 2C). 

#### 4.1.2. Essential Oils 

**Figure 3:**
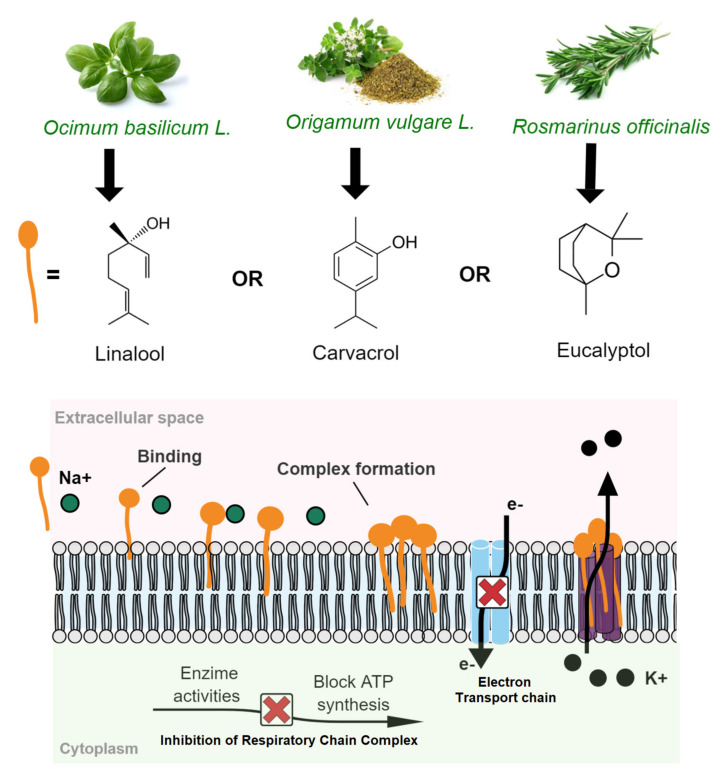
Antimicrobial action triggered by monoterpenoid compounds from basil, oregano, and rosemary essential oils: damaging cell membrane integrity and inhibiting a mitochondrial respiratory chain complex.

The oxygenated terpenes sucg as linalool, carvacrol, and eucalyptol are bioactive compounds present in primary contents at basil EO (*O. basilicum*), oregano (*O. vulgare*), and rosemary (*R. officinalis*) EOs, respectively, with antimicrobial activity. The antimicrobial mechanism of linalool was proved to be associated with damaging cell membrane integrity-triggered by membrane permeability (by interactions with phospholipids), resulting in nucleic acid releasing and membrane depolarization, thus resulting in cell death by measures of releasing nucleic acids and membrane potential [38]. Furthermore, these monoterpenoids’ effects on the cell membrane can also inhibit the mitochondrial respiratory chain complex-triggered by proton pump dysfunction and intracellular ATP depletion [45,50,51]. Figure 3 shows the antimicrobial mechanisms of action associated with the destruction of membrane cell integrity triggered by monoterpenoid compounds from *O. basilicum*, *O. vulgare*, and *R. officinalis* essential oils.

#### 4.1.3. Polysaccharides, Polypeptides, and Enzymes 

Polysaccharides such as chitin, chitosan (CS), and chitooligosaccharides (COS) are derivatives of chitin deacetylation, the main compound found in crustacean’s wastes, and they have been widely studied as natural antimicrobials in food purposes against fungi, Gram negative, and Gram positive bacteria [52,53,54]. Moreover, chitosan is a biopolymer that has also been proposed as a food packaging material with several advantages (e.g., biodegradability, bioactivity, biocompatibility) over traditional petroleum-based plastic films [55]. Among the antimicrobial mechanisms of chitosan, the most acceptable is associated with electrostatic interactions between the positively charged chitosan (and negative residues) and the negatively charged microbial cell membrane, which results in two types of interference: (i) changing the properties of membrane wall permeability, inducing osmotic imbalance; and (ii) hydrolyzing peptidoglycan in the cell wall, causing spillage of intracellular electrolytes and essential nutrients [40]. Natamycin and nisin are other examples of natural macromolecules resulting from bacterial fermentation widely used as food bio preservatives [56]. Natamycin (or pimaricin) is a polyene macrolide used effectively only against yeasts and molds due to its binding to ergosterol in the fungal cell membrane [45]. Nisin is a polypeptide bacteriocin derived from lactic acid bacteria (LAB). The non-efficacy of natamycin against bacteria was explained in terms of the bacteria membrane’s lack of sterols. Similarly, to saponins, the nisin antimicrobial action is generally due to the generation of pores in the membrane, impairing cell wall biosynthesis by specific lipid II binding interactions inhibiting microbial growth [57]. Both have been studied relative to cheese packaging with starch edible films or additives during cheesemaking [58,59,60].

Similarly, lysozymes [22,61,62,63] were used in active edible packaging/coatings to inhibit Gram positive bacteria in cheese. Lysozyme (naturally occurring enzyme by bodily secretions) and poly-lysine (a biocompatible cationic polymer of amino acids L-lysine or D-lysine and derived from natural bacterial fermentation) showed high antibacterial action due to their capacity to hydrolyze the peptidoglycans’ cell wall. Recently, the antimicrobial mechanism of ε-Poly-lysine on the bacterial cell was investigated. The leakage of intracellular materials and intracellular enzymes was proved through measurements of the loss of soluble proteins inhibition of respiratory metabolism via the Embden-Meyerhof-Parnas glycolytic pathway [64]. 

**Figure 4:**
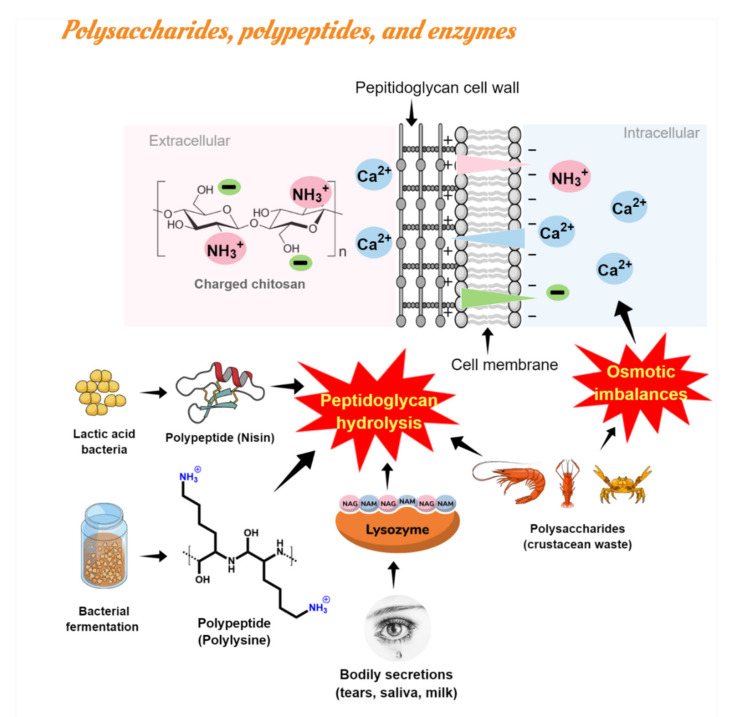
Antimicrobial action of polysaccharides, polypeptides, and lysozyme: membrane permeability alteration and hydrolysis of peptidoglycans in the microbial cell wall by electrostatic interactions.

Figure 4 summarizes the most common antimicrobial mechanism linked with natural antimicrobials such as polysaccharides (CS, chitin), polypeptides (nisin, poly-lysine), and lysozyme. 

### 4.2. Microbiological, Shelf Life, and Quality Parameters of Cheese Preservative by Natural Ingredients and Combined Treatments 

#### 4.2.1. Natural Antimicrobials in Cheese Preparation to Extend the Shelf Life

Table 2 presents the inhibition effects and shelf-life data overviewed relative to spices and plants in different product forms (i.e., polysaccharide compounds, plant extract, essential oils, and liposomes) used as natural antimicrobial alternatives to traditional additives in terms of preserving cheeses obtained by in vitro and in situ assays. What is interesting about the data in this table is that almost all natural antimicrobials reduced the *Staphylococcus aureus* count on the surface of Iranian white brined cheese and was at least 6.56% higher than the control cheese with an excellent shelf-life (up to 75 days of storage), as in the case of 15 µL/100 mL of Cumin EO (*Cuminum cyminum*), incorporated with the probiotic *Lactobacillus acidophilus* at 0.5% *w*/*v* during cheese manufacturing [65]. However, despite the outstanding results of 30 µL/100 mL of Cumin EO (CR: 51.36% higher than control), the doses recommended were 15 µL/100 mL once the sensory analysis and organoleptic evaluation were determined to be superior and did not show significant differences in pH values between them [65]. Afterward, the reduction in *L. monocytogenes* counts on Iranian white cheese by Pennyroyal EO (*Mentha pulegium*) at a dosage of 0.015% was 24% higher than the control cheese at the end of 60 days of storage [66]. Despite the better result of antimicrobial effect promoted by the dosage of 0.03% EO (CR of 49% higher), the cheese with 0.015% EO presented more extensive overall acceptability in terms of sensory quality [66]. The shelf life results can be associated with the high contents of monoterpenoids in these oils, as the major compounds identified by Gas chromatography-mass spectrometry (GC-MS) in Cumin EO were cuminaldehyde (29.02%), α-terpinene-7-al (20.70%), and gamma-terpinene (12.94%) [65]. By comparison, the major compounds identified in Pennyroyal EO were pulegone (36.68%), piperitenone (16.88%), and 1,8 cineole (14.58%) [66]. 

Likewise, the ginger EO (*Zingiber officinale*), also rich in monoterpenes and sesquiterpenes, was mainly composed of α-zingiberene (16.1%) and geranial (14.4%), followed by Z-citral (9.2%), β-cedrene (8.6%), geranial acetate (8.4%), α-pinene (6.1%), α-curcumene (5.3%), and α-farnesene (4.4%) [72]. Thus, preliminary results with 12% (*v*/*v*) ginger EO demonstrated an in vitro bactericidal effect against pathogenic strains (*Pseudomonas aeruginosa*, *Salmonella enterica* ser. Typhimurium, *S. aureus*, *E. coli O157:H7*, *L. monocytogenes*, and *S. aureus*) once the MBC/MIC was two with an inhibition zone ranging from 13–37 mm [72]. The quality parameters of cheese were not evaluated.

The ethanolic extract of dry cedar leaves (*M. oleifera*) was rich in nutritional and phenolic components and was suggested as supplemental nutrition and preservative agent to extend the shelf life of cream cheese. The amount of 4.00 mg of cedar ethanolic extract (95%) showed significant in vitro antimicrobial action against pathogenic strains (e.g., *Bacillus cereus*, *Bacillus subtilis*, *S. aureus*, *P. aeruginosa*, *L. monocytogenes*, *Escherichia coli*, *and Salmonella*) with inhibition zone diameter ranging from 15–22 mm. At the same, the concentration per milliliter of milk in the preparation of cream cheese, the coliforms, yeasts, and molds growth inhibition in cream cheese was 100% once any count was observed during the 4 weeks of storage, contrary to the negative control sample [42]. For quality, besides the addition of *M. oleifera* extract improved sensory parameters of taste and flavor, the color changed due to dietary fibers’ presence.

The fennel (*Foeniculum vulgare* Mill) is a distribution plant in Central Europe and the Mediterranean with antimicrobial and antioxidant properties [67,76]. Due to these properties, Caleja et al. (2015) [67] reported a useful in vitro antimicrobial activity of fennel phenolic-enriched extract against *B. cereus*, *S.* *enterica* ser. Typhimurium, *Aspergillus niger*, and *Aspergillus versicolor* by MIC values ranging from 0.02 to 0.75 mg/mL, with the *S. enterica* ser. Typhimurium and *B. cereus* are the most sensitive to fennel extract by the MIC values of 0.035 and 0.02. Furthermore, cottage cheese preparation with fennel extract improved antioxidant properties 62.96% greater than the control cheese and up to 14 days of storage without degradation signals [67]. 

Black cumin (*Bunium persicum*) is a typical plant in Afghanistan and Iran’s dry temperate regions [77] that was chemically characterized and presented cuminaldehyde (11.4%) as the most abundant bioactive compound in its oily seeds [68], which are frequently associated with good antioxidant and antibacterial properties. Thus, Ehsani et al. (2016) [68] evaluated EO’s antimicrobial capacity from the seeds of black cumin against *L. monocytogenes* and *E. coli O157: H7* on Iranian White Cheese. At the end of the 45 day storage, the *L. monocytogenes* and *E. coli* count reduction was 7–9% and 6–9% higher than the control, respectively, by low EO doses from 1–2% (*w*/*v*) [68]. Furthermore, the black cumin EO showed higher sensory quality attribute scores (e.g., color, texture, flavor, odor, and general acceptability) than the control.

The pink pepper EO (*Schinus terebinthifolius*) is widely distributed in South America (Brazil, Paraguay, and Argentina) and has been reported with high in vitro and in situ antimicrobial activity in several food matrices. An inhibition zone ranging from 39 to 97 mm was reported by in vitro antibacterial assay against *B. cereus* at a MIC of 0.85 mg/mL-EO from mature fruits, and it was better than green fruits. Its biopreservative potential in situ by low doses (0.7–2% of EO-mature pulp) was also demonstrated for fresh Minas-type cheese against *L. monocytogenes* extending the shelf life up to 30 days of storage, with a count reduction 11–18% higher than the control cheese [70]. Despite this, the authors mentioned its potentially undesirable effects on sensory quality. 

As discussed before, basil oil (*O. basilicum* L.) is another essential oil rich in terpenes and phenolics (e.g., linalool and estragole) that stands out as a preservative due to its antimicrobial and antioxidant properties [43,44]. Likewise, LAB can also produce substances (e.g., enzymes, bioactive lipids and peptides, vitamins, and exopolysaccharides) that inhibit pathogenic microorganisms and improve dairy products’ protection and functional properties, in addition to its role in milk fermentation [78]. Thus, its characteristics might explain the synergistic effect of 5% basil oil and different LAB strains as antimicrobial in unripened goat cheese during 120 h of storage by 100% inhibition of *Enterobacteriaceae* growth compared to the control cheese [44]. Moreover, there was no significant change in the product’s pH; the texture with the addition of basil and LAB (*L. brevis*) was 1.3% lower when compared to the control cheese; as for colors, the L* values of cheese containing basil-LAB was lower, while a * and b * was higher than the control cheese; and for sensory quality, cheese containing basil-LAB had higher acceptability (5 points on the hedonic scale) than the control cheese (4 points) [44].

The identification of carvacrol (80.9%) and ρ-cymene (7.7%) as the significant constituents of thyme EO (*Thymus algeriensis*) seems to be a primary determining factor of its antioxidant and tremendous broad-spectrum in vitro antibacterial and antifungal activity reported by Bukvicky and colleagues (2018). These authors related expressive values of MIC ranging from 0.03 to 0.09 mg/mL, and MBC ranged from 0.05 to 0.15 mg/mL against Gram negative and Gram positive bacteria (*S. aureus*, *S. enterica* ser. Typhimurium, *E. coli*, *P. aeruginosa*, *L. monocytogenes*, *Micrococcus flavus*, and *B. cereus*), as well as the impressive MIC of 0.01 mg/mL against *Aspergillus* spp., *Trichoderma* spp., and *Penicillium* spp. [71]. In terms of cheese shelf life, this study found a reduction in *Penicillium aurantiogriseum* contamination incidence (65% higher than control sample) on sliced soft cheese after 30 days of storage at 4 °C by the use of thyme EO at a concentration of 25 µL [71]. Concerning sensory quality, the 15 µL EO improved color and texture parameters but did not stands out in terms of flavor compared to the control cheese [71]. 

The intense preservative action of ε-poly-lysine against *L. monocytogenes* on the Grana Padano cheddar cheese surface at a low dosage (from 0.05 to 0.2 mg/mL) was 30–100% higher than control cheese after 15 days of storage at 4 °C and 25 °C [64]. 

In addition to ginger (*Z. officinalis)*, garlic (*Allium sativum* L.) has also stood out in terms of several biological properties such as antimicrobials relative to preserving cheese, although the sensory quality can be affected for both level and type of spice. Recently, Salih et al. (2019) [73] evaluated the antimicrobial effect of garlic and ginger powders at concentrations ranging from 2% to 6% on white cheese storage for 14 days at 4 °C. The authors reported that any count of Coliforms and *Salmonella* was detected for the lowest content (2%) of booth garlic and ginger powder during the period of storage. By contrast, garlic powder showed a more potent action than ginger powder against fungi and yeasts since, when applied at 2–6% concentration, its count reduction was 58.6–79.3% higher than the control cheese [73]. Regarding the sensory quality, in addition to the 2% ginger-cheese having better results in terms of overall acceptability than the control cheese, all samples with garlic powder had greater overall acceptability (taste, flavor, and texture) by the consumer (as well as pH) than the control or ginger powder [73]. However, all treatments showed lower color scores than the control [73]. 

Recently, the addition of combining terpene-rich oregano EO (0.03 µL/mL) and rosemary oil (1.32 µL/mL) during cheesemaking was suggested as a natural preservative to fresh cheese during refrigerated storage once the synergistic effect between them for inhibiting *E. coli* O157: H7 growth was 68% higher than the control cheese during 21 days of storage [45]. Furthermore, the quality parameters were also improved once the cheese containing both EO showed lower hardness and better results of softness and chewiness and similar values for L * and b * in color compared to the control cheese. Until the end of 21 days of storage, the authors detected eucalyptol, camphor, and α-pinene, which produced a refreshing aroma, minty flavor, and softness to fresh cheese in addition to explaining the shelf life results obtained [45]. 

The application of the gaseous phase of Lemon leaf EO (*Citrus limon* var *Pompia*) in ricotta Salata cheese slices showed a tremendous antimicrobial activity against *L. monocytogenes* by a MIC of 0.086 µL/cm^3^, which is probably related to phytochemicals screened as linalyl acetate, limonene, and two Citral isomers—neral and geranial [75]. Thus, low doses of gaseous phase-lemon EO, ranging from 0.05 to 1 mL, resulted in a decrease in the *L. monocytogenes* count of 21–60% higher than the control Ricotta Salata cheese after 30 days refrigerated storage (at 5 °C). In addition to that, the natural antimicrobial did not cause a significant difference in the LAB and total mesophilic bacteria (TMC) [75]. 

The evaluation of green propolis ethanol extract (EEP) (*Apis mallifera*) as a cheese preservative was motivated by its tremendous in vitro bactericide and fungicide effects against several yeasts and bacteria (*Staphylococcus*, *Bacillus*, *Enterococcus*, *Corynebacterium*, and *Proteus* spp.) by MFC/MIC ranging from 1 to 2.2 and MBC/MIC equal 2, respectively, with the most sensitive being *S. cerevisiae* (MFC = 0.63%) and the most resistant fungi being *Candida parapsilosis* (MFC = 5%) [74]. Thus, a dosage from 1.25% (*w*/*w*) EEP was capable of completely inhibiting *Staphylococcus saprophyticus*, and the *Staphylococcus equorum* count after 24–28 h, 37 °C, as well as from 1.25% or 2.5% (*w*/*w*) completely inhibited *Yarrowia lipolytica* or *Debaryomyces hansenii* after 48–72 h (for yeasts) at 37 °C on the surface of Gorgonzola-type cheese. However, only 5% EPP was suggested as a promising concentration once it showed a sensory quality (i.e., taste, odor, and overall) similar to the control cheese, and 10% EPP was worse than the control [74]. 

Despite the potential antimicrobial effect of several essential oils in extending the shelf life of cheeses, certain oils have low solubility, photosensitivity, and high volatility and can negatively interfere in the milk matrix by reducing its sensory quality [79]. Thus, these disadvantages contribute to studying these oils in the encapsulated form in order to prevent adverse effects on the cheese’s quality or physicochemical properties. Despite lemongrass oil (LO) (*Cymbopogon citratus*) decreasing the physical and sensory quality of cheese at the end of 15 days of storage, LO encapsulated in a liposome (1 mL·LO/100 g) promoted a count reduction in *L. monocytogenes* on Kerrygold Cheddar 59% that was higher than free LO and the control, without affecting the color surface, texture, and sensory quality (e.g., aroma and taste) of cheese at the end of storage [69]. 

#### 4.2.2. Essential Oils Amplifying the Preservative Action of Sodium Salts

Table 3 displays the synergism’s microbial growth inhibition effect between natural antimicrobials and sodium salts relative to extending the shelf life of cheese. Relative to *L. monocytogenes* growth, one interesting finding in this review is that the addition of essential oils in milk during cheese preparation can minimize *L. monocytogenes* resistance to salt and its high tolerability of high salt concentrations [80]. However, the efficacy of 2% spearmint EO (*Mentha spicata*) incorporated into fresh ewe’s milk against *L. monocytogenes* on Lighvan Cheese was improved by the storage temperature (14 °C) and water salt concentration (15%) during 60 days [80]. The physical-chemical and sensory parameters were not evaluated in this study.

A strong relationship between the bioactive compounds of thyme oil (e.g., α-Pinene, 1,8-cineole, and camphor) or rosemary oil (e.g., thymol and carvacrol) and antimicrobial activity has been reported in the literature [45,71]. The effectiveness of 1% (*w*/*w*) rosemary EO and 1% (*w*/*w*) thyme EO against *L. monocytogenes* in low-fat Mozzarella cheese was 8.3% and 15.4%, respectively, and these are higher than the control cheese; the synergistic effect between them was proved to be 21% higher than the control, and the combination of them with 0.2% sodium diacetate preservative was even better once it was 52% higher than the control cheese [81]. Despite any oil treatment influencing the color of freshly shredded cheese, the flavor was impaired by sensory quality analysis [81]. Interestingly, the authors suggested that if the cheese was used to prepare baked products (e.g., pizza), the adverse effects on flavor could be reduced once the oil’s volatile compounds could be eliminated by oven heating. 

#### 4.2.3. Natural AntimicrobialsinActive Coatings/Nanopackaging for Preserving Cheese

Closer inspection of Table 4 shows natural antimicrobials as active ingredients of coating and packaging with a broad-spectrum microbial inhibition higher than control cheese.

If the ratio MBC/MIC ≤ 4, the effect was considered bactericidal, but the effect was defined as bacteriostatic if MBC/MIC > 4 [82]. Thus, most of the studies overviewed revealed higher bactericidal and bacteriostatic effects with prolonged shelf life (>60 days of storage) at low doses of the antimicrobial compound. The addition of pink pepper EO as a bioactive ingredient into film packaging at a concentration of 5.45 mg/cm² showed 100% antimicrobial activity against *S. aureus* and *L. monocytogenes* in sliced mozzarella cheese during the 12 days of storage, once no count was detected [82]. The acetate cellulose film was chosen for packaging due to its capacity to form films at low temperatures, avoiding EO’s volatilization during polymer film processing [82]. Moreover, the in situ assay also demonstrated the affinity between EO’s non-polar components with lipids of cheese, allowing EO migration from the film to cheese. The authors suggested that it could benefit the application of active packaging by direct contact [82]. This strategy is promising for eliminating the disadvantages of undesirable effects on the product’s sensory quality when the direct addition of EO occurs during cheesemaking [70]. 

A novel nanopackaging of a bio-nanocomposite film made from 0.92% (*w*/*w*) chitosan, 0.92% (*w*/*w*) cellulose gum, and 2–8% (*w*/*w*) zinc oxide nanoparticles (CS/CMC/ZnO) enhanced the shelf life of Egyptian white soft cheese completely inhibited with coliforms, TBC, yeast, and molds and reduced pathogenic strain contamination (*S. aureus*, *Bacillus* spp., *P. aeruginosa*, *E. coli*, L. *monocytogenes*, *C. albicans*, and *A. niger*) with IZ values ranging from 8 to 15 mm during 30 days of storage at 7 °C [85]. Regarding cheese quality, contrary to the control cheese, the characteristic color of fresh cheese was preserved, and the moisture, pH, and titrable acidity were preserved without molds during storage period in cheese packaged by bionanocomposite film [85].

Preliminary results of in vitro antibacterial effect of ginger EO reported MIC of 2.3 µL/mL and MBC of 4.7 µL/mL for *L. monocytogenes* and *E. coli* O157:H7 as the most sensitive MO, as well as CR: 20–40% *L. monocytogenes* by micro-atmosphere (at 0.12 to 0.35 µL/cm^3^) due to the action of volatiles from EO [72]. Ginger EO as a nanoscale antimicrobial encapsulates in the protein ultrafine fibers (12% *v*/*v*; EO/polymer), purposing active packaging of the Minas-fresh cheese, and reduced *L. monocytogenes* contamination after 12 days of storage with the count reduced to 10% higher than the control. The cheese’s physical-chemical and sensory parameters were not evaluated in the study in question [72].

Pomegranate peel extract (PPE) in zein films has become an alternative to Himalayan cheese (Kalari) packaging. In the study of Mushtaq et al. (2018) [86], the PPE concentrations of 25, 50, and 75 mg/g of a film-forming solution were evaluated for their in vitro antibacterial activity of IZ until 20 mm (101 to 123% higher than the control) against *E. coli*, *P. perfringens*, *Micrococcus luteus*, *Enterococci faecalis*, *S. aureus*, *Proteus vulgaris*, and *S. enterica* ser. Typhi. Regarding shelf life on day 21 of storage by in situ assay using the Kalari as cheese model, the PPE concentration range of 25–75 mg/g showed a reduction in the microbial counts of 54–73% and 71–100% for total bacterial count and yeasts and molds, respectively, which are higher compared to the control cheese [86]. The sensory analysis performed during the tenth day of storage on cheese containing 75 mg/g PPE was preferred in the evaluated items (i.e., appearance, flavor, aroma, bitterness, and overall acceptability) compared to the control cheese [86].

Another critical finding was other natural antimicrobials from the protist kingdom such as sodium alginate or alginic acid, a polysaccharide obtained from brown algae, and its association with essential oils with consumer appreciation in sensory quality. Sodium alginate as a coating additive containing 3% (*w*/*v*) potassium sorbate inhibited the *Pseudomonas* spp. and other psychrotrophic microorganisms growth in fresh mozzarella cheese, with an efficacy 12% higher than the control cheese during 8 days of storage (at 8 ± 1 °C), preserving the sensory quality compared to the control that was refused after 4 days [84]. Recently, 0.2% sodium alginate’s effectiveness as a coating additive for Béja Sicilian cheese preservative was confirmed with the advantages of replacing the chemical additive with 3% *Pimpinella saxifrage* EO as the active ingredient [87]. The most exciting finding was that despite the strategy improving bacterial stability by inhibiting three-gram positive (*B. cereus*, *M. luteus*, and *L. monocytogenes*) and three-gram negative (*E. coli*, *P. aeruginosa*, and *S. enterica* ser. Typhimurium) bacteria by bactericidal and bacteriostatic action, the weight loss, color, pH, and oxidative stability were also preserved without flavor impairment during 60 days of refrigerated storage [87]. Additionally, Ksouda et al. (2019) [87] also assessed *Pimpinella saxifrage* EO’s acute toxicity, showing that doses of 250 and 500 mg/kg had no harmful effects on the mice model. The presence of the flavoring compound anethole, eugenol isomers, and p-anisaldehyde as main compounds in *P. saxifrage* EO might explain its great results beneficial to cheese preservation. 

The effect of lactic acid (LA) and chitooligosaccharide (COS) as active ingredients for edible-whey protein isolate film was compared to PVA-commercial coating containing natamycin as an active ingredient applied on the surface of Portuguese cheese during 15 days of storage [83]. While natamycin showed the highest efficacy against yeasts and molds, the edible coating formulated with LA and COS showed a potency of 98% and 100% count reduction higher than the control against *Pseudomonas* and *Staphylococcus* species [83]. Natamycin also has been used as an active ingredient in active bio-packaging to preserve cheese during storage, despite being effective only against fungi. For example, natamycin was an active ingredient of active packaging made from tapioca starch film (at 9.25 mg/dm^2^ film) and preserved Port Salut cheese against *S. cerevisiae* with an efficacy 62% higher than samples using natamycin applied by spraying technique, during 216 h of storage at 25 °C [59]. Afterward, recent investigations by Seydim and colleagues (2020) evaluating the effect of oregano 2% EO, garlic EO, nisin, or natamycin as active ingredients of whey protein isolate film (WPI) and natamycin observed the highest reduction count (until 20% higher than control) against *Penicillium* spp., while nisin had the highest reduction count (until 25% higher than control) against *L. monocytogenes* during 15 days of storage. On the other hand, the films containing oregano and garlic EO stand out against *E. coli* O157:H7 strains, with 40% efficacy higher than the control at day 15 of storage [88]. However, the investigations citing natamycin and nisin as active ingredients of bio-packaging for preserving cheese did not analyze the impact on cheese quality. 

#### 4.2.4. Combined Methods: Natural Antimicrobials and Packaging Conditions in Non-Thermal Treatments 

Table 5 displays the effect of combined treatment on extending the shelf life of cheese and microbial count reduction percentage. What is striking about this table’s data is that while lysozyme/Na_2_-EDTA salt does not affect the inhibition growth of *Pseudomonas* species when burrata cheese was packaged in air, the combination of them with modified-atmosphere packaging in the *Pseudomonas* spp. Showed that count reduction was at least 22–39% higher than the control during 7 days of storage [89]. Unfortunately, both the MAP and lysozyme used affected the sensory attributes such as texture and overall acceptability of cheese [89]. 

Likewise, the pine needle extract (*Cedrus deodora*), rich in acidic and phenolic compounds, was also suggested as a natural preservative for cheese due to improved oxidation stability and storage quality of low-fat Kalari cheese treated with 2.5–5% pine needles extract, dried, and aerobically packaged in low-density polyethylene bags [90]. From the minimum concentration, the authors have already observed favorable microbiological results during the 28 days of storage at 1 ± 4 °C once coliform growth was not detected in any sample (the milk for cheese manufacturing was heat-treated at 90 °C), while an antifungal effect at least 17% higher than the control cheese was reported for samples treated with extracts against Yeasts and Molds. Furthermore, this research also reported a reduction until 17% and 7% higher than the control cheese in the count of psychrophilic bacteria and total plate count, respectively [90]. The pine needles extract improved the Kalari cheese physicochemical parameters once both doses of 2.5% or 5% at the pH of 4.35 or 4.18, respectively, were below the control cheese pH (4.71) at the end of the 28 days of storage; [90]. Furthermore, sensory quality parameters (e.g., appearance, color, flavor, texture, sourness, and overall acceptability) have been preserved [90].

First, unlike the sodium benzoate in Table 1, citric acid as an active ingredient of tapioca flour packaging (10 g/100 g) treated with pulsed light against *L. innocua* on sliced cheddar cheese (Table 5) was 36% higher than the control during storage; however, the product quality (e.g., pH, moisture, and mechanical properties) changed after 7 days of refrigerated storage [26]. Nevertheless, the adjustment of citric acid content is promising once the antioxidant effect prevented cheddar cheese color instability due to lipid oxidation [26].

## 5. Conclusions and Outlook

Firstly, in this review, we discussed the main microorganism contaminating cheese (e.g., Gram positive and Gram negative bacteria, coliforms, yeasts, and molds) as the most crucial factor in cheese preservation and how unsuitable traditional chemical additives (e.g., potassium sorbate, sodium benzoate, and sodium chloride) can be once potential harmful impacts on human health by their use as a food preservative had been reported as the reason for concern by several researchers. Thus, alternative options with natural antimicrobial agents that are safer and healthier in addition to the significant antimicrobial efficacy to extend shelf life in cheese have received researchers’ significant attention in the last two decades, focusing their strategies by using essential oils, plant extract, and spices. Understanding the cellular mechanisms of action of phytochemicals from natural origins was crucial to predict how a given natural compound can act on a given group of microorganisms. Among different microbial metabolic signaling pathways reported as natural compounds, the most sensitive has been related to alterations in membrane permeability, cell wall structure and metabolism alterations, and cell depolarization. Some examples with primary and secondary metabolites were discussed for terpenes, phenolics, aminoglycosides, enzymes, and naturally occurring polymers such as polysaccharides and polypeptides. In the second part of this review, the antimicrobial effects of in vitro/in situ assay, shelf life, and cheese quality were assessed compared to a cheese control using several efforts purposing cheese preservatives with healthier attempts: essential oils, plant extracts, and spices added during cheese preparation; essential oils combined with sodium salts; active coatings and active packaging using biomaterials; and combined treatments such as antimicrobial agents, packaging conditions, and non-thermal methods of processing technologies. 

The papers reviewed indicate that the all-natural compounds studied showed an antimicrobial effect higher than the control cheese by low dosages, and most of them demonstrated a broad-spectrum antimicrobial action. While commercial coating of natamycin only has fungicide action, oregano and garlic EO incorporated in edible protein film could improve the shelf life of cheese and protect it against fungi and bacteria. By contrast, although the potential side effects on health exists, some chemical additives had no effect on shelf life than compared to the control cheese.

Despite the potential antimicrobial effect of several essential oils in the extending shelf life of cheeses (until >75 days of storage), certain oils negatively interfered in the milk matrix, reducing its final properties. Thus, these disadvantages contributed to studying these oils in the nano encapsulated form to prevent adverse effects on cheese’s quality or physicochemical properties. Essential oils also amplified the antimicrobial action of sodium salts commonly used by the cheese industry as a flavor and preservative to resistant and tolerable bacteria. 

Combined treatment strategies such as natural antimicrobial and non-thermal processing technology can overcome the challenge of some bacteria (e.g., *L. monocytogenes* and *E. coli* O157:H7) that are able to survive in the brines used to store cheeses, which cannot be heat sterilized to preserve the yeasts and LAB essential for normal cheese maturation. Unfortunately, we note that antimicrobial agents associated with non-thermal processing technologies as pulsed light and MAP still compromise the cheese sensory quality. Contrarily, antimicrobials attempts in active nanopackaging made from bio-nanocomposite stood out in this review as a promising strategy due to the improved shelf life and its ability to preserve the physicochemical parameters of Egyptian white soft cheese after storage. 

Our study review makes several contributions to the current literature and cheese industry. The great potential of natural antimicrobials in controlling cheese contamination it has already been understood, and the significant limitation of the current literature is the absence of an evaluation of the impacts on the sensory and physicochemical quality of the cheeses by using the various alternatives that have been successful in preventing microbial contamination in the cheeses reported here. Thus, our review has not established if the required concentration of the natural antimicrobial for inhibiting microbial growth is economically viable for large-scale industrial applications. The possibility for separating natural antimicrobials into groups according to the best suitability for certain types of cheese can still be evaluated. However, in the case of essential oil as a preservative for Mozzarella cheese, if the cheese was used to prepare baked products, the adverse effects on flavor could be reduced once the oil’s volatile compounds can be eliminated by oven heating. Taken together, the analysis of evidence from this review where most of the attempts reported better overall preserved cheese quality suggests that the use of active coating and active packaging with edible biopolymers is perhaps the most promising attempt. Recent advances showed that essential oil as a nanoscale antimicrobial (e.g., nano capsules and ultrafine fibers) promoted bacteriostatic and bactericidal effects.

Moreover, the sensory quality and physicochemical properties can be preserved without flavor impairment in a prolonged shelf life (until 60 days of refrigerated storage) when EO was an active ingredient for active bio-packaging. We also consider the synergistic effect of both active ingredients and biopolymer of packaging (e.g., starch, proteins, alginic acid, and cellulose) as a sustainable food preservative with fewer side effects on health. Future opportunities were identified for investigation as well as for application in the food industry in terms of the toxicological effects of certain insights for preserving cheeses reported here. 

## Figures and Tables

**Table 1:** Traditional chemical additives to preserving cheese.

Chemical Additive	Treatment	Study Model	Dose	Target MO	Growth Inhibition	Ref.
Potassium sorbate	Additive during manufacturing	White cheese (curd)	0.1% *w/w*	*L. monocytogenes*	Without effect ^A^	[25]
		White cheese (brine)	0.1% *w/w*		Without effect ^A^	
		White cheese (curd)	0.1% *w/w*	*S. aureus*	Without effect ^A^	
		White cheese (brine)	0.1% *w*/*w*		Without effect ^A^	
	Additive	In vitro	0.15%	*L. monocytogenes*	CR: 100% ^B^	[27]
	PS-SA-coating	Low-moisture Mozzarella cheese	1% *w*/*v*	*Total viable counts*	CR: 3.23% ^C^	[28]
	PS-SA-coating and MAP3 packaging	Low-moisture Mozzarella cheese	1% *w*/*v*	*Total viable counts*	CR: 3.23% ^C^	[28]
Sodium benzoate	SB-starch film	Cheddar cheese slices	0.001% *w*/*w*	*L. innocua*	Without effect ^D^	[26]
	SB-starch film and pulsed light		+6.14 J/cm^2^	*L. innocua*	CR: 28% ^D^	

PS: potassium sorbate; SA: sodium alginate; MAP3: modified atmosphere packaged (50% CO_2_ and 50% N); SB: sodium benzoate; MO: microorganism; CR: microbial count reduction -% CFU relative to control; ^A^: Made from pasteurized milk and stored at 4 °C for 60 days; ^B^: after 60 days stored at 4 °C, pH 5.6; ^C^: after 78 days of storage at 4 °C; ^D^: after 30 days of refrigerated storage.

**Table 2:** Spices, plant extracts, or essential oils as natural antimicrobial alternatives to traditional additives for extending the shelf life of cheese.

Specie	Common Name (Form)	In Vitro/In Situ Assay	Target MO	Dose	Growth Inhibition	Storage Time	Ref.
*Cuminum cyminum*	Cumin (EO)	Iranian white brined cheese	*S. aureus*	15 µL/100 mL	CR: 6.56%	75 days	[65]
				30 µL/100 mL	CR: 51.36%		
*Foeniculum vulgare* Mill	Fennel (water extract)	In vitro	*B. cereus*	MIC: 0.02 mg/mL	-	N/A	[67]
			*S. enterica* ser. Typhimurium	MIC: 0.035 mg/mL	-		
			*A. niger*	MIC: 0.2 mg/mL	-		
			*A. versicolor*	MIC: 0.75 mg/mL	-		
*Mentha pulegium*	Pennyroyal (EO)	Iranian White cheese	*L. monocytogenes*	0.015%	CR: 24.39%	60 days	[66]
				0.03%	CR: 48.78%		
*Bunium persicum*	Black Cumin (EO)	Iranian White cheese	*L. monocytogenes*	1–2%	CR: 7–9%	>45 days	[68]
			*E. coli* O157:H7		CR: 6–9%		
*Cymbopogon citratus*	Lemongrass oil	Kerrygold cheddar	*L. monocytogenes*	1 mL·LO/100 g	CR: 0%	15 days (4 °C)	[69]
	LO-entrapped liposomes				CR: 58%		
*Schinus terebinthifolius* Raddi	Pink pepper (EO-mature fruits)	In vitro	*B. cereus*	MIC: 0.85 mg/mL	IZ: 39.97 mm	N/A	[70]
		Minas-type fresh cheese	*L. monocytogenes*	0.7–2%	CR: 11–18%	30 days	
*Ocimum basilicum* L.	Basil-LAB bioproducts fermentation	Goat milk curd cheese	*Enterobacteriaceae*	5%	100% (N/D)	24–120 h	[44]
*Moringa oleífera*	Cedar (dry leaves extract)	In vitro	Pathogenic strains	4.00 mg/mL·milk	IZ: 15–22 mm	N/A	[42]
		Cream cheese	Coliforms, Molds, and Yeasts		100% (N/D)	4 weeks	
*Thymus algeriensis*	Thyme (EO)	In vitro antibacterial	*S. aureus*	MIC: 0.08 mg/mL	-	N/A	[71]
			*S. enterica* ser. Typhimurium	MIC: 0.09 mg/mL	-		
			*E. coli*	MIC: 0.09 mg/mL	-		
			*P. aeruginosa*	MIC: 0.05 mg/mL	-		
			*L. monocytogenes*	MIC: 0.04 mg/mL	-		
			*M. flavus*	MIC: 0.03 mg/mL	-		
			*B. cereus*	MIC: 0.04 mg/mL	-		
		In vitro antifungal	*Aspergillus* spp.	MIC: 0.01 mg/mL	-	N/A	
			*Trichoderma* spp.	MIC: 0.01 mg/mL	-		
			*Penicillium* spp.	MIC: 0.01 mg/mL	-		
		Sliced soft cheese	*Penicillium aurantiogriseum*	25 µL	CI: 66%	30 days (4 °C)	
*Zingiber officinale*	Ginger (EO)	In vitro antibacterial activity	*P. aeruginosa*	MBC/MIC = 2	IZ: 13 mm	N/A	[72]
			*S. enterica* ser. *Typhimurium*	MBC/MIC = 2	IZ: 15 mm		
			*S. aureus*	MBC/MIC = 2	IZ: 19 mm		
			*E. coli* O157:H7	MBC/MIC = 2	IZ: 19 mm		
			*L. monocytogenes*	MBC/MIC = 2	IZ: 37 mm		
MO fermentation	ε-poly-lysine solution (0.1 mg/m)	Grana Padano cheddar cheese	*L. monocytogenes*	MIC: 0.05–0.2 mg/L	CR:30–100%	15 days, (4 and 25 °C)	[64]
*Allium sativum* L.	Garlic powder	White cheese	Mold and Yeast	6%	CR:58.6–79.3% ^A^	15 days (4 °C)	[73]
			Coliforms, *Salmonella*	2–6%	100% (N/D) ^A^		
*Zingiber officinale* Roscoe	Ginger powder		Mold and Yeast	2–6%	CR: 51.7% ^A^		
			Coliforms, *Salmonella*	2–6%	100% (N/D) ^A^		
*Origanum vulgare* L. + *Rosmarinus officinalis*	Oregano EO + Rosemary EO	Fresh cheese	*E. coli* O157:H7	0.03 (oregano) and 1.32 (rosemary) µL/mL	CR: 68%	21 days	[45]
*Apis mallifera*	Green propolis (extract)	Gorgonzola-type cheese	*Y. lipolytica*	1.25%	100%	24–48 h (37 °C)	[74]
			*D. hansenii*	2.50%	100%		
			*S. saprophyticus*	1.25%	100%	48–72 h (25 °C)	
			*S. equorum*	1.25%	100%		
		In vitro antifungal	*S. cerevisieae*	MFC/MIC = 2	-	N/A	
			*D. hansenii*	MFC/MIC = 1	-		
			*C. parapsilosis*	MFC/MIC = 2.2	-		
		In vitro antibacterial	*B. cereus*	MBC/MIC = 1.9	-		
			*S. saprophyticus*	MBC/MIC = 2	-		
			*S. equorum*	MBC/MIC = 2	-		
*Citrus limon* var *pompia*	Lemon leaf (EO-gaseous phase)	Ricotta Salata	*L. monocytogenes*	0.5–1 mL	CR: 21–65%	30 days (5 °C)	[75]

MO: microorganisms; EO: essential oil; MIC: minimum inhibitory concentration; N/D: not detected; -: not reported; N/A: not applicable; LAB: lactic acid bacteria; CR: count reduction by % CFU decreasing relative to control; LO: lemongrass oil; LAB: lactic acid bacteria; ^A^: on cheese processed; IZ: inhibitory zone; CI: contamination incidence; MFC: minimum fungicide concentration; MBC: minimum bactericidal concentration.

**Table 3:** Synergism between essential oils and sodium salts for extending the shelf life of cheese.

Specie	Common Name (Form)	Salt	Cheese Model	Target MO	Doses	Inhibition Effect	Storage Time	References
*Mentha spicata*	Spearmint EO	Sodium chloride	Lighvan cheese	*L. monocytogenes*	2% EO + 12% NaCl	RIZ: 48%	60 days (4 °C)	[80]
					2% EO + 15% NaCl	RIZ: 22%		
					2% EO + 12% NaCl	RIZ: 50%	60 days (14 °C)	
					2% EO + 15% NaCl	RIZ: 55%		
*Rosemarinus officinalis*	Rosemary EO	Sodium diacetate	Low-fat Mozzarella	*L. monocytogenes*	1%	CR: 8.3%	20 days (4 °C)	[81]
*Thymus spicata*	Thyme EO				1%	CR: 15.4%		
*R. officinalis* and *T. spicata*	Rosemary and Thyme (EO)				1%	CR: 26.1%		
					+0.2% SDA	CR: 52.5%		

MO: microorganisms; EO: essential oils; NaCl: sodium chloride; CR: count reduction by % CFU decreasing relative to control; SDA: sodium diacetate; RIZ: % of inhibitory zone relative to control.

**Table 4:** Active coating/packaging technology loading natural antimicrobials as a green preservative method extending cheese’s shelf life.

Specie/Origin	Common Name (Form)	Packaging Material	In Vitro/In Situ	Target MO	Dose	Inhibition Effect	Storage Time	Ref.
*Schinus terebinthifolius*	Pink pepper (EO)	Cellulose acetate film	Sliced mozzarella cheese	*S. aureus*	5.45 g/cm^2^ (EO in film	100%-not detected	0–12 days	[82]
				*L. monocytogenes*		100%-not detected		
LAB fermentation	Lactic acid (compound)	Edible-Whey protein isolate film	Portuguese cheese	*Staphylococcus* spp.	CA: 6 g/L and COS: 20 g/L	CR: 100%	15 days	[83]
				*Pseudomonas* spp.		CR: 98%		
Crustacean by-products	COS			Yeast and mold		CR: 28%		
				*Pseudomonas* spp.		CR: 100%		
Actinobacteria	Natamycin	PVA-Commercial coating		Yeast and mold	2.5 g/L	CR: ~43%		
Actinobacteria	Natamycin (compound)	Tapioca starch film	Port Salut cheese	*S. cerevisiae*	9.25 mg/dm^2^ of film	CR: 62%	216 h (25 °C)	[59]
Seaweed	Sodium alginate solution (2% *w*/*v*)	Potassium sorbate (3% *w*/*v*)	Fresh Mozzarella cheese	*Pseudomonas* spp.	3% (*w*/*v*)	CR: 12%	8 days (8 ± 1 °C)	[84]
				*Enterobacteriaceae*	2% (*w*/*v*)	CR: 12%		
Crustacean by-products	Chitosan (compound)	CS/CMC/2–8% ZnO film bio packaging	Egyptian white cheese	TBC, Coliforms, Yeasts and molds	0.92% (*w*/*w*; CH/film)	100%	30 days (7 °C)	[85]
Cellulose-cotton, wood	CMC (gum)			Pathogenic strains	0.92% (*w*/*w*; CMC/film)	IZ: 8–15 mm		
*Punica granatum*	Pomegranate (peel extract)	Zein films	Himalayan cheese (Kalari)	TBC	25–75 mg/g	54–73%	15 days	[86]
				Yeast and Mold	25–75 mg/g	71–100%		
			In vitro antibacterial activity	*E. coli*	25 mg/g	RIZ: 123%	N/A	
				*P. perfringens*	75 mg/g	RIZ: 118%		
				*M. luteus*	75 mg/g	RIZ: 114%		
				*E. faecalis*	50 mg/g	RIZ: 115%		
				*S. aureus*	50 mg/g	RIZ: 107%		
				*P. vulgaris*	50 mg/g	RIZ: 114%		
				*S. enterica* ser. Typhi	50 mg/g	RIZ: 101%		
*Zingiber officinale*	Ginger (EO)	-	In vitro antibacterial activity	*E. coli* 0157:H7	MIC: 2.3 µL/mL	IZ:13 mm	N/A	[72]
				*L. monocytogenes*	MBC: 2.3 µL/mL	IZ: 37 mm	N/A	
		Ginger EO Encapsulation in PUF	Antibacterial by micro-atmosphere	*L. monocytogenes*	12% (*v*/*v*)	CR: 20.9–43.5%	N/A	
			Minas-type fresh cheese	*L. monocytogenes*	12% (*v*/*v*)	~10%	12 days (4 °C)	
*Pimpinella saxifraga*	Burnet saxifrage EO (3%)	Sodium alginate as coating additive(0.2%)	Béja Sicilian cheese	*S. enterica* ser. Typhimurium	MBC/MIC = 1.9	RIZ: 19%	60 days	[87]
				*B. cereus*	MBC/MIC = 2	RIZ: 36%		
				*M. luteus*	MBC/MIC = 2	RIZ: 39%		
				*E. coli*	MBC/MIC = 4	RIZ: 50%		
				*P. aeruginosa*	MBC/MIC = 4	RIZ: 39%		
				*L. monocytogenes*	MBC/MIC = 8	RIZ: 52%		
*Origanum vulgare* L.	Oregano (EO)	Whey protein isolate film	Sliced Kasar cheese	*Penicillium* spp.	136.6 mg/g (2% *w*/*v*)	CR: 15%	15 days	[88]
				*E. coli* O157:H7		CR: 40%		
*Allium sativum* L.	Garlic (EO)			*Penicillium* spp.		CR: 10%		
				*E. coli* O157: H7		CR: 22%		
Actinobacteria	Natamycin (compound)	Whey protein isolate film	Sliced Kasar cheese	*Penicillium* spp.	2% (*w*/*v*)	CR: 20%	15 days	[88]
LAB	Nisin (compound)	Whey protein isolate film	Sliced Kasar cheese	*L. monocytogenes*	2% (*w*/*v*)	CR: 25%		[88]

MO: microorganisms; PVA: polyvinyl acetate; CS/CMC/ZnO: chitosan/carboxymethylcellulose/zinc oxide bionanocomposites; CA: citric acid; COS: chitooligosaccharide; CR: count reduction by % CFU decreasing relative to control; LDPE: low-density polyethylene; EO: essential oils; TBC: total bacterial count; % RIZ: inhibitory zone relative to control; N/A: not applicable; MIC: minimum inhibitory concentration; MBC: minimum bactericidal concentration; PUF: protein ultrafine fiber.

**Table 5:** Combined treatments for extending the shelf life of cheese as a green alternative to traditional additives: antimicrobial agents, processing technology, and packaging conditions.

Specie/Origin	Antimicrobial	Combined Treatment	In Vitro/In Situ Assay	Target MO	Dose	Inhibition Effect	Storage Time	Ref.
Animal secretions	Lysozyme	Active coating and air packaging	Burrata cheese	*Pseudomonas* spp.	250 mg/Kg	Without effect	7 days	[89]
Chemical additive	EDTA disodium salt				50 mM			
Animal secretions	Lysozyme	Active coating and MAP packaging		*Pseudomonas* spp.	150–500 mg/Kg	CR: 22–39%		
Chemical additive	EDTA disodium salt				50 mM			
*Cedrus deodora* (Roxb.) Loud	Pine needles (extract)	Manufacturing additive and Aerobically packaging	Low-fat Kalari	Total plate	2.5–5%	CR: 14–17%	30 days (1 ± 4 °C)	[90]
				Psychrophilic	2.5–5%	CR: 3–7%		
				Yeast and Mould	2.5–5%	CR: 17–39%		
Citrus fruits, sugar cane	Citric acid (compound)	Active packaging and pulsed light	Sliced cheddar cheese	*L. innocua*	0.001% (*w*/*w*)	CR: 28%	At the first 5 days	[26]
					+6.14 J/cm^2^ pulsed light	CR: 36%		

MO: microorganisms; CA: citric acid; CR: count reduction by %CFU decreasing relative to control; MAP: modified-atmosphere packaging; SA: sodium alginate; PS: potassium sorbate.

## List of Abbreviations/Acronyms

ADHD	Attention Deficit Hyperactivity Disorder
BITC	Benzyl isothiocyanates
CA	Citric acid
CFU	Colony-forming Units
CR	Microbial count reduction
CS	Chitosan
CS/CMC/ZnO	Chitosan/carboxymethylcellulose/zinc oxide bionanocomposites
CMC	Carboxymethylcellulose
COS	Chitooligosaccharide
CVD	Cardiovascular disease
EDTA	Ethylenediamine tetraacetic acid
EEP	Propolis ethanol extract
EO	Essential oil
FDA	Food and Drug Administration
GC-MS	Gas chromatography-mass spectrometry
GRAS	Generally recognized as safe
IZ	Inhibitory zone
LA	Lactic acid
LAB	Lactic acid bacteria
LDPE	Low-density polyethylene
LO	Lemongrass oil
MAP	Modified-atmosphere packaging
MBC	Minimum bactericidal concentration
MFC	Minimum fungicide concentration
MIC	Minimum inhibitory concentration
MO	Microorganism
NaCl	Sodium chloride
PPE	Pomegranate peel extract
PS	Potassium sorbate
PUF	Protein ultrafine fiber
PVA	Polyvinyl acetate
RIZ	Inhibitory zone relative to control
SA	Sodium alginate
SB	Sodium benzoate
SDA	Sodium diacetate
TBC	Total bacterial count
TMC	Total mesophilic bacteria
USFDA	The United States Food and Drug Administration
WHO	World Health Organization
WPI	Whey protein isolate
ZnO	Zinc oxide nanoparticles
